# The cognitive impacts of Chinese pinyin learning on English-speaking elementary students in a bilingual educational setting

**DOI:** 10.3389/fpsyg.2025.1626414

**Published:** 2025-10-27

**Authors:** Jingzhi Liu, Hao Bai

**Affiliations:** ^1^School of International Education, Nanjing University of the Arts, Nanjing, Jiangsu, China; ^2^College of Information Engineering, Hainan Vocational University of Science and Technology, Haikou, Hainan, China; ^3^Brisight (Hainan) Technology and Development Co., Ltd., Haikou, Hainan, China

**Keywords:** Chinese pinyin, second language acquisition, phonological awareness, cross-linguistic interference, bilingual elementary learners, biliteracy development

## Abstract

This study investigates the effects of limited Chinese pinyin learning on American elementary school students' language performance, with particular emphasis on potential cross-linguistic interference and the benefits of pinyin learning. Participants from two schools, one receiving Chinese instruction including pinyin (experimental group) and one with no Chinese instruction (control group), in first and fourth grades were assessed on measures of Nonverbal Ability (NA), Phonological Awareness (PA), the Peabody Picture Vocabulary Test (PPVT-4), Chinese Fluency Test (CFT), Commonly Mispronounced Chinese Words Read Aloud Test (CMCT), and Commonly Mispronounced English Words Read Aloud Test (CMET). Results indicated that the modest exposure to pinyin, delivered as five 30-min sessions per three weeks, did not significantly interfere with the acquisition of English vocabulary, as evidenced by comparable performance on PPVT-4 and CMET across groups. In first grade, CMCT was positively correlated with NA, PA, and CMET, while in fourth grade, significant correlations were observed only with CMET and CFT. Furthermore, error analyses revealed that the majority of mis- pronunciations were attributable to intrinsic properties of the English language rather than to negative transfer from pinyin instruction. These differential patterns suggest that a developmental shift in how Chinese pinyin interacts with students' cognitive and linguistic abilities across grade levels, the most effective period for introducing pinyin instruction appears to be around the first grade, when phonological systems are still developing, while by fourth grade, when these systems are largely consolidated, the cognitive benefits of pinyin learning become less direct, although early pinyin skills then appear to support vocabulary acquisition.

## 1 Introduction

The role of pinyin in Chinese language instruction has attracted considerable attention. In his influential work Language Issues (1980), Zhao Yuanren argued that foreign language learning comprises three essential components—pronunciation, grammar, and vocabulary—and that instruction should proceed sequentially through these stages (Zhao, 1980). This sequential approach is consistent with theories in second language acquisition that emphasize the importance of establishing a strong foundation in phonetic competence to support the later acquisition of grammatical structures and vocabulary (Ellis, 1999; Spada and Lightbown, 2006). Consequently, mastery of pinyin, as the cornerstone of phonetic instruction, is critical for learners of Chinese as a second language. Evidence indicates that proficiency in pinyin enhances pronunciation accuracy and overall language competence (Bassetti, 2007).

In adult language learning, Chinese pinyin is regarded as an auxiliary tool for developing phonetic skills (Lü, 2017). Adult learners are typically introduced to pinyin at the beginner level to establish a foundational understanding of Chinese phonetics and to build a support system that facilitates further language acquisition (Cai and Liu, 2011). Moreover, pinyin instruction assists in the teaching of Chinese tones and fosters autonomous study skills (Chang, 2018). However, the effects of pinyin instruction differ between adult and child learners. Adults, having developed a mature first language system, are generally better equipped to adapt to an additional phonetic framework. In contrast, for children who are native English speakers—their English phonological system still under development, the introduction of a second phonetic system raises several critical questions. Similar multicomponent patterns have been reported for Chinese reading fluency, where phonological awareness (PA) remains a strong concurrent predictor (Zhang et al., 2021), further underscoring the importance of examining how pinyin relates to children's phonological development. For example, does learning pinyin interfere with the acquisition of spoken English vocabulary? If interference occurs, what is its extent, and when might it diminish? Conversely, might the native English phonological system hinder the acquisition of pinyin? These questions are central to optimizing models of Chinese as a second language instruction in English-dominant contexts (Koda, 2005; Cummins, 2000).

A related issue concerns whether elementary school children should be taught Chinese pinyin and, if so, when and how this instruction should be delivered. Some educators advocate postponing pinyin instruction until after the second grade to prevent disruption of the early development of the native language's phonological and orthographic system (Wang, 2021). Others maintain that pinyin does not significantly interfere with native language skills; even if minor interference occurs, it is quickly overcome, suggesting that pinyin should be introduced from the beginning to leverage its pedagogical benefits (Lü, 2017). A third viewpoint argues against the use of pinyin entirely, positing that since Chinese characters form the authentic writing system of Chinese, introducing pinyin may confuse learners by conflating their native language with Chinese, and therefore Chinese instruction should begin directly with characters (Lin et al., 2020; Yan, 2005). This debate can be distilled into two primary issues: determining whether pinyin instruction is necessary for children learning Chinese as a second language, and examining whether pinyin instruction interferes with the acquisition of spoken English vocabulary, whether the native orthographic system hinders pinyin learning, and what benefits pinyin instruction might provide (Koda, 2005; Cummins, 2000; Bialystok, 2001).

From a theoretical perspective, the necessity of pinyin instruction for children acquiring Chinese as a second language can also be examined through developmental models of biliteracy. According to Cummins's (1992) common underlying proficiency hypothesis, developing biliteracy—defined as proficiency in both the native and second language writing systems—leads to changes in children's emergent literacy awareness at both foundational and surface levels. In practical terms, the impact of biliteracy is far-reaching, influencing literacy development in both the short and long term. Findings from bilingual cognitive development further suggest that exposure to multiple language systems can enhance cognitive control and executive functions (Bialystok, 2001), which may facilitate learners' ability to integrate diverse orthographic and phonetic systems. Early explanations for certain errors in biliteracy acquisition attributed them to interference from the native language. For instance, Spanish orthographic rules require the insertion of an *e* after an *s* when followed by the sounds [t], [p], or [k] (e.g., in *estrella* and *español*), which led Spanish-speaking children learning to write English to produce errors such as *estop* or *esky*. Such errors were once seen as evidence of native language interference in second language acquisition, leading to a preference for bilingual education over biliteracy programs in elementary foreign language instruction. More recent evidence, however, demonstrates that instruction supporting both written and oral language forms is essential; without written symbols, bilingual instruction is markedly less effective (Baker, 2011). Furthermore, occasional confusion during biliteracy learning reflects children's active use of native language knowledge in constructing second language competence, a productive application of first language skills (Valdés and Castellón, 2010; Uro and Lai, 2019). Typically, this influence manifests as the dominant language affecting the acquisition of the less dominant language, rather than the reverse (Bialystok and Craik, 2010).

Although previous research has examined bilingual phonological awareness and the role of alphabetic scripts in second language learning, relatively little is known about how limited and early exposure to pinyin affects the cognitive development of English-speaking elementary students, leaving a gap in understanding how pinyin functions as a scaffold in early bilingual literacy development. To anchor these questions, the present study employed several standardized and researcher-developed measures. In addition to the Nonverbal Ability test (NA), the Phonological Awareness test (PA), and the Peabody Picture Vocabulary Test (PPVT-4), we introduced three additional tools. The Commonly Mispronounced English Words Test (CMET) and the Commonly Mispronounced Chinese Pinyin Test (CMCT) were designed to assess children's pronunciation when reading English words and Chinese pinyin, respectively—items that are prone to mispronunciation due to overgeneralization of grapheme–phoneme correspondences, partial overlap between English and Chinese phonological rules, or significant cross-linguistic differences. In addition, the Chinese Fluency Test (CFT) was administered to evaluate students' ability to process Chinese vocabulary and syntax through immediate translation tasks, thereby reflecting their overall oral proficiency in Chinese. In this study, CMET was used to examine potential transfer effects from pinyin to English, CMCT to investigate the influence of English phonology on early pinyin learning, and CFT to capture broader outcomes of Chinese language development. By employing a longitudinal cross-lagged design, the present study provides new evidence on the developmental dynamics of pinyin learning, particularly the shift from phonological to lexical correlates across grade levels. In doing so, it extends current theories of bilingual education by showing that even minimal exposure to a phonetic system outside the native language can produce measurable, stage-specific benefits without compromising first-language literacy. This highlight instead the importance of timing and developmental readiness in second language instruction.

In summary, the role of pinyin in Chinese language instruction is multifaceted. While pinyin serves as a vital tool for enhancing phonetic competence and supporting language development among adult learners, its introduction in early instruction for children, particularly those from English-speaking backgrounds, raises critical issues regarding potential interference effects and the optimal timing and method of instruction. This study aims to address these issues by examining the effects of pinyin instruction on Chinese language acquisition and exploring its interaction with native language literacy development.

## 2 Research background and literature review

Recent investigations of bilingual children across diverse language pairs, such as Spanish–English, French–English, and Chinese–English, indicate that bilingual experiences not only facilitate the simultaneous acquisition of both languages but also enhance overall linguistic and nonverbal cognitive abilities (Bialystok, 2007; Marian and Shook, 2012; Barac and Bialystok, 2012; Bice and Kroll, 2021). The process by which children overcome instances of “confusion” in a second language appears to promote the refinement of grapheme–phoneme correspondence, thereby strengthening literacy awareness. In addition, research exploring the underlying mechanisms of these cognitive benefits suggests that bilingual experience bolsters central executive functioning, yielding advantages in both language-specific and domain-general cognitive tasks as early as childhood (Barac and Bialystok, 2011).

Thomas and Collier (1997) posit that bilingual children who attain academic fluency exhibit cognitive advantages relative to their monolingual peers, ultimately achieving higher levels of academic performance (Ovando et al., 2003). Buckwalter and Lo (2002) further contend that during early language emergence, the simultaneous acquisition of two writing systems—exemplified by the concurrent development of Chinese and English—does not result in mutual interference; rather, the two systems may reinforce each other in reading and writing, thereby deepening overall linguistic understanding. Large-scale data on Chinese–English bilinguals demonstrate that early biliteracy experience boosts EF while causing only transient L1 lags (Yin et al., 2022).

In overseas contexts, the process of Chinese language acquisition among children diverges markedly from that of native Chinese-speaking children. Typically, native learners acquire a phonological–semantic mapping through immersion in the target language before gradually learning the orthographic system in a sequential manner. In contrast, children learning Chinese as a foreign language must simultaneously acquire character form, pronunciation, and meaning, which imposes a considerably heavier cognitive load. Even when instructional emphasis is primarily placed on oral communication rather than literacy, the lack of an immersive phonological environment accentuates the importance of pinyin as a supportive tool. Pinyin accuracy can serve as an early behavioral marker of subsequent reading success (Ma et al., 2020). Given the inherent complexity of Chinese characters, direct instruction using characters without the intermediary support of pinyin would compel learners to master form, sound, and meaning concurrently—a challenge of substantial magnitude. Thus, the pinyin system plays an indispensable role in facilitating Chinese language acquisition in non-native settings, arguably even more so than for adult learners. Without the support of pinyin, the efficiency of Chinese language learning is significantly reduced. Consequently, in overseas environments, the incorporation of pinyin instruction is essential for establishing a robust phonological framework that underpins effective oral language development (Chai and Bao, 2023; Lü, 2017; Zhang S.-Z. et al., 2020; Liu, 2023).

In summary, the acquisition of Chinese pinyin is crucial for foreign children learning Chinese. During the learning process, even if bilingual children exhibit superficial con- fusion between two language systems, such confusion does not necessarily impede the development of their native language (Zhao, 2022; Chen et al., 2019; Mok et al., 2018). Rather, this phenomenon may reflect the productive application of native language knowledge to second language acquisition. Furthermore, simultaneous exposure to two languages and writing systems may enhance children's sensitivity to linguistic structures and bolster their fundamental language abilities (Zhao, 2022; Xie et al., 2022). A recent meta-analysis estimates PA to explain 20% of the variance in L2 Chinese reading across 31 samples (Chen and Zhao, 2022), underscoring its centrality.

Given the critical role of pinyin instruction, several issues merit further investigation: (1) Does the acquisition of Chinese pinyin interfere with the development of the native language's phonological or orthographic system, and if so, what is the extent and duration of such interference (Zhang and MacWhinney, 2023; Cui, 2014; Pan et al., 2023)? (2) In cases where interference is minimal or absent, does the native phonological system affect the acquisition of pinyin (Bassetti, 2006; Zhang J. et al., 2020; Hayes-Harb and Barrios, 2021; Xiao et al., 2020; Chang, 2018)? (3) What specific benefits does pinyin instruction confer in terms of overall language proficiency, particularly with respect to oral language development (Lü, 2017; Zhang et al., 2019; Harvey and Brooks, 2022; Shi, 2019)?

Collectively, these considerations underscore the necessity of continued research into the role of pinyin instruction in optimizing Chinese language learning outcomes for foreign children. Beyond PA, morphological awareness also differentially supports Chinese word reading (Liu et al., 2022), an avenue for future work.

## 3 Research experiment method

### 3.1 Participants

A total of 64 students from two elementary schools in the United States participated in the study. All participants were native English speakers from a central region of the country. The experimental group was drawn from School G, which enrolls students from Kindergarten through Grade 4. At School G, Chinese had been the only compulsory foreign language course since two years prior to the implementation of this experiment; classes are held five times every three weeks, with each session lasting 30 min—comparable in duration to the school's art and music classes. At the time of testing, Grade 1 students had received one semester of Chinese instruction focused primarily on oral communication, with pinyin used as the principal written symbol system. Although Grade 4 students had been learning Chinese since Grade 2, they had not been introduced to pinyin during the first two years. Formal pinyin instruction commenced only after the start of the experiment, so that at the time of testing, both Grade 1 and Grade 4 students had received one semester of pinyin instruction.

The control group was selected from School N, which has a student population similar in size to School G; however, students at School N do not study any foreign language. Specifically, the experimental group from School G consisted of 32 Chinese- learning students (16 from Grade 1 and 16 from Grade 4, each from one intact class), and the control group from School N comprised 32 non-Chinese-learning students (16 from Grade 1 and 16 from Grade 4, each from one intact class).

To strengthen comparability between groups, Schools G and N were selected from the same public school district and serve demographically similar, predominantly middle-income catchment areas. According to district administrative records, the schools did not differ materially in socioeconomic status (proxied by free/reduced-price lunch eligibility) or in ethnic composition. Importantly, neither school offered any foreign language program prior to the introduction of Mandarin at School G, minimizing the possibility of prior exposure to other languages. Both schools follow the same district curriculum and assessment policies. Together with the baseline equivalence observed on cognitive and language measures reported in the Results, these features provide a principled basis for matching the experimental and control schools and help support the study's internal validity.

To further contextualize potential transfer effects, demographic data were collected regarding participants' language exposure at home. All students came from predominantly monolingual English-speaking households. Students at N School had never been exposed to Chinese, either formally or informally, prior to the study. At School G, Chinese had been the only compulsory foreign language course since two years prior to the implementation of this experiment; however, the participating students had not received any Chinese instruction before enrolling in the school's Chinese-as-a-second-language program. Occasional exposure to other languages in community or media contexts was reported by fewer than 5% of families, but these cases were incidental and not systematic. Therefore, the sample can be characterized as English-dominant monolingual, minimizing confounds related to prior multilingual experience.

### 3.2 Measures

#### 3.2.1 Nonverbal ability (NA)

Nonverbal reasoning was assessed using the Matrix Reasoning subtest of the Wechsler Abbreviated Scale of Intelligence (WASI) (Wechsler, 2011). In this task, children were presented with an incomplete matrix composed of abstract figures and instructed to select the shape from an array of six options that correctly completed the matrix.

#### 3.2.2 Phonological awareness (PA)

Phonological awareness was assessed using three subtests.

In Subtest 1, the *Rhyme Choice* task evaluates awareness of rhyme endings. Each of ten sets begins with a target word, and participants are asked to select, from three subsequent words, the one that rhymes with the target (e.g., given the target word star, the options include scar, tell, and seat).

Subtest 2 assesses syllable integration and the ability to substitute the initial consonant. In the *Substitute Initial Consonant* task, each of ten sets starts with a target word; participants are instructed to replace the initial sound of the target word with a specified alternative (e.g., replacing the initial sound of top with /h/ to form hop).

Subtest 3 examines awareness of final consonants using the *Final Consonant Same* task. Similar to Subtest 1, each of ten sets begins with a target word, and participants must choose, from three subsequent words, the one that shares the same final consonant sound as the target word. Unlike Subtest 1, however, the examiner provides a corresponding picture for the target word in Subtest 3. Related research suggests that presenting a visual cue for the target word can reduce the memory load on participants (Roberts, 2017; van Nooijen et al., 2024). For example, when the target word is worm, participants are provided with a simple diagram of a worm on an A4 sheet, along with options as come, put, and plane.

#### 3.2.3 English vocabulary (PPVT-4)

The Peabody Picture Vocabulary Test (Fourth Edition; PPVT-4) was employed to evaluate the participants' English vocabulary. In this assessment, students were required to select the picture that best represented a spoken word from an array of images. Administration of the test was discontinued for a participant once eight or more errors were made within a single set, in accordance with standardized termination criteria (Dunn and Dunn, 2007; Goriot et al., 2021).

#### 3.2.4 Commonly mispronounced English words read aloud test (CMET)

Participants were asked to read aloud a series of printed English words. The test comprised 30 words that were selected based on their frequency in the English lexicon and their susceptibility to interference from Chinese phonological patterns. The first 15 words were chosen because their pronunciation at the letter (alphabetic) level is particularly vulnerable to the influence of Chinese pinyin instruction, while the subsequent 15 words were selected due to their potential to be affected at the syllabic level (Khanal et al., 2021; Lin, 2007).

#### 3.2.5 Chinese fluency test (CFT)

The Chinese Fluency Test (CFT) used a translation task where participants listened to Chinese words, phrases, and sentences then immediately translated them into English. Grade 1 students translated 24 words, three phrases, and three sentences. Grade 4 students completed the same 24 words but with expanded subtests of six phrases and six sentences. This task measures their ability to process Chinese vocabulary and syntax, reflecting overall fluency.

#### 3.2.6 Commonly mispronounced Chinese pinyin read aloud test (CMCT)

Participants were instructed to read aloud a series of printed Chinese pinyin syllables. The test items were selected from syllables that children had previously encountered in their Chinese language instruction and that are particularly prone to being confused with English pronunciation. A total of 35 words were included in the test.

Importantly, none of these items were annotated with tone markers in order to avoid providing cues that might lead to pronunciation differences relative to English. This omission was intended to ensure that the test specifically measured the influence of Chinese pinyin learning on the pronunciation of the syllables, without the confounding effects of tonal information (Bassetti, 2007; Dong, 2023).

### 3.3 Instructor qualifications and instructional fidelity

All Chinese language classes at School G were taught by certified teachers holding graduate degrees in Teaching Chinese as a Foreign Language or related fields, each with at least three years of experience in bilingual elementary settings. To ensure instructional fidelity, teachers followed a standardized curriculum guide that specified lesson objectives, instructional materials, and pacing. Periodic fidelity checks were conducted through classroom observations by the program coordinator and cross-review of lesson plans to confirm consistency across grades. Furthermore, instructional content was aligned with recognized standards for early Chinese as a foreign language education (e.g., ACTFL performance descriptors and introductory HSK-level, YCT-level benchmarks). the state value-added assessment system, and the state Educator Acceleration Model. These alignments ensured that instructional outcomes were compatible with both international benchmarks and local accountability systems. Collectively, these measures helped minimize variability due to instructor differences and enhanced the reliability of the instructional intervention.

## 4 Experimental procedures

At School G, six tests were administered twice with a 4-month interval between cycles. In each cycle, students first completed the CMET, PA, PPVT-4, and NA tests in order, with about a 2-min break between each test (Dunn and Dunn, 2007; North and Schneider, 1998). Then, after an interval of more than two days, they took the remaining CMCT and CFT tests. In contrast, School N did not administered in the two tests directly related to Chinese proficiency, instead, only administered the first four tests once, following the same procedure as School G's initial session. Each child was assessed individually in a one-on-one session at their respective school (Mackey and Gass, 2015). Prior to participation, written informed consent was obtained from the children's legal guardians, and age-appropriate assent was also obtained from the children themselves. Researchers explained the study procedures in simple language and emphasized that participation was voluntary and could be discontinued at any time.

Scoring was done by two experienced Chinese language teachers. For the NA and PPVT-4 tests, scores were assigned based on the test manuals (Dunn and Dunn, 2007; Mackey and Gass, 2015): NA's maximum score was 28 for Grade 1 and 32 for Grade 4, while PPVT-4 had a maximum of 160 for both grades. In the PA test, each correct answer earned 1 point, while incorrect responses received 0 points. For the CMET, each word was scored from 0 to 3 points (0 for no response or more than two errors, 1 for two errors, 2 for one vowel or consonant error, and 3 for a fully correct pronunciation). Similarly, each pinyin syllable in the CMCT was scored on a 0-to-3 scale: 0 for no response or both components incorrect, 1 for only one component being correct, 2 when one component was correct and the other approximated the target, and 3 for complete accuracy. In the CFT, points were allocated as 2 per word, 3 per phrase, and 4 per sentence, with final scores reflecting overall performance (Dunn and Dunn, 2007; North and Schneider, 1998). It should be noted that the CMET, CMCT, and CFT were specifically developed for this study. Item selection followed two main criteria: (i) high frequency in children's vocabulary, and (ii) prior inclusion in the instructional content already covered by the participating students. This ensured that the tests reflected learned material rather than unfamiliar items. In addition, item selection for the CMET and CMCT also considered susceptibility to mispronunciation due to overlap or conflict between English and Chinese phonological rules. To ensure content validity, all items were reviewed by two experienced Chinese language teachers. Inter-rater reliability for scoring was high (Cohen's κ>0.85 across both tests). A pilot administration with 12 non-participant students confirmed that the test instructions were easily understood and that the scoring rubric produced consistent results. Taken together, these steps provide preliminary evidence that the CMET and CMCT are reliable tools for capturing cross-linguistic pronunciation challenges in early bilingual contexts, as well as for assessing students' capacity to process Chinese vocabulary and syntactic structures as indicators of overall language proficiency.

## 5 Experimental results

### 5.1 Analysis of differences in assessment scores

In the first assessment session, the reliability of all subtest measures across both schools exceeded 0.8 (Cronbach's α>0.81). Detailed test scores for each subtest are presented in Table 1.

**Table 1:** Mean (standard deviation) scores by grade and school.

**Measure**	**G school**		**N school**	
	**First grade**	**Fourth grade**	**First grade**	**Fourth grade**
NA	13.50 (3.81)	22.06 (5.45)	10.38 (2.22)	21.06 (5.23)
PA	93.54 (7.25)	98.33 (3.44)	83.13 (14.06)	97.08 (3.19)
PPVT-4	119.88 (12.78)	118.06 (16.27)	119.75 (14.22)	120.44 (12.19)
CMET	85.07 (10.70)	95.21 (3.00)	85.42 (9.86)	95.14 (2.95)
CFT	43.39 (8.16)	38.75 (13.86)	–	–
CMCT	71.07 (11.03)	80.48 (6.28)	–	–

IQ and PPVT-4 represent average raw scores; all other scores represent average accuracy percentages (%).

In the analysis of first-grade participants, an ANOVA was conducted on the NA scores from both schools. The interaction effect between school and NA was not statistically significant (*p* > 0.05). After controlling for NA differences, the mean PA score for first-grade participants at G School was 93.255, compared to 83.412 for those at N School; this difference was statistically significant (*p* < 0.05), indicating that G School participants outperformed their N School counterparts in PA. In contrast, the differences in PPVT-4 and CMET scores between the two schools for first graders were not statistically significant (*p* > 0.05).

For fourth-grade participants, ANOVA of NA scores similarly showed that the interaction between school and NA was not statistically significant (*p* > 0.05). After controlling for NA, no significant differences were observed between the schools in terms of PA (*p* > 0.05), PPVT-4 (*p* > 0.05), or CMET (*p* > 0.05).

These results suggest that the cognitive levels and English proficiency of children at the two schools were essentially homogeneous. Moreover, the lack of significant differences in CMET scores across both grades indicates that one semester of Chinese pinyin instruction did not adversely affect the pronunciation of English words that are prone to confusion. Notably, first-grade students at G School with Chinese pinyin experience demonstrated significantly higher PA scores than their counterparts at N School with no Chinese learning experience, whereas no such difference was observed in fourth grade.

**Figure 1:**
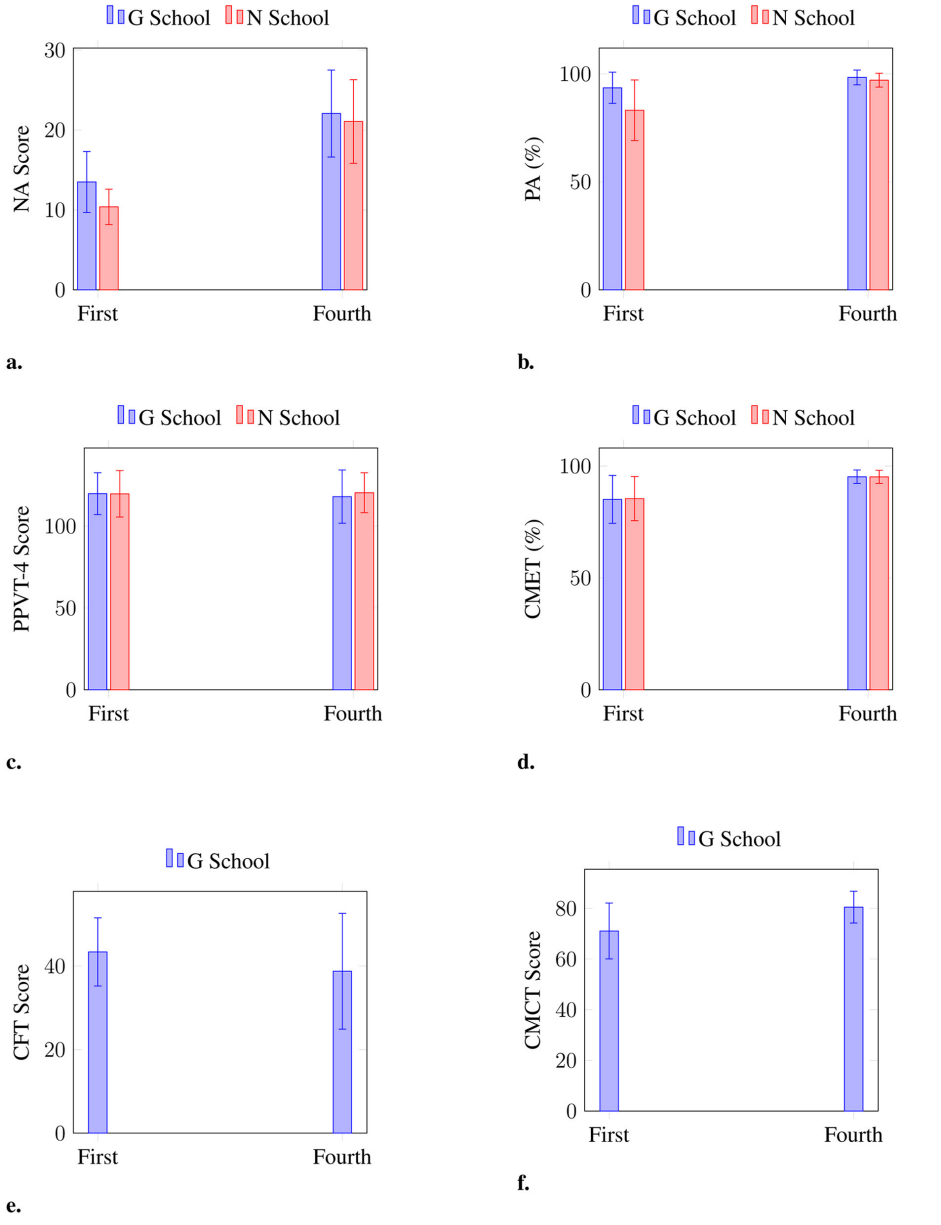
Mean (±SD) scores for each subtest by grade and school. Note that CFT and CMCT were administered only to participants from G School. **(a)** Nonverbal ability (NA). **(b)** Phonological awareness (PA). **(c)** Peabody picture vocabulary test (PPVT-4). **(d)** Commonly mispronounced English words read aloud test (CMET). **(e)** Chinese Fluency Test (CFT). **(f)** Commonly mispronounced Chinese pinyin read aloud test (CMCT).

Figure 1 provides a visual representation of these findings. For example, Figure 1a illustrates the NA scores for first-grade participants, while Figure 1b displays the corresponding PA scores. These sub-figures further support the statistical outcomes described above.

Table 2 presents the measurement scores after School G continued studying Chinese for an additional four months, along with the paired *t*-test results comparing the two testing sessions. The paired *t*-test reflects the children's progress. According to Table 2, both grades showed significant improvements in PA, CMET, and in CFT. However, PPVT-4 improved significantly only in first grade—not in fourth grade—while performance on confusable Chinese pinyin words showed no significant progress in either grade.

**Table 2:** Paired *t*-test results for cycle-1 and cycle-2 scores in school G.

**Measure**	**Grade 1 Mean (SD)**	***t***	**Grade 4 Mean (SD)**	***t***
PPVT-4	123.75 (11.45)	−3.156^**^	118.19 (13.73)	−0.104
PA	98.33 (2.98)	−3.523^**^	99.38 (2.50)	−2.611^*^
CMET	90.28 (7.13)	−3.127^**^	96.60 (3.31)	−2.825^*^
CMCT	71.79 (13.10)	−0.493	81.07 (6.93)	−0.837
CFT	50.97 (15.08)	−2.204^*^	55.31 (19.32)	−3.007^**^

Values in parentheses are standard deviations. ^*^p < 0.05, ^**^p < 0.01.

**Figure 2:**
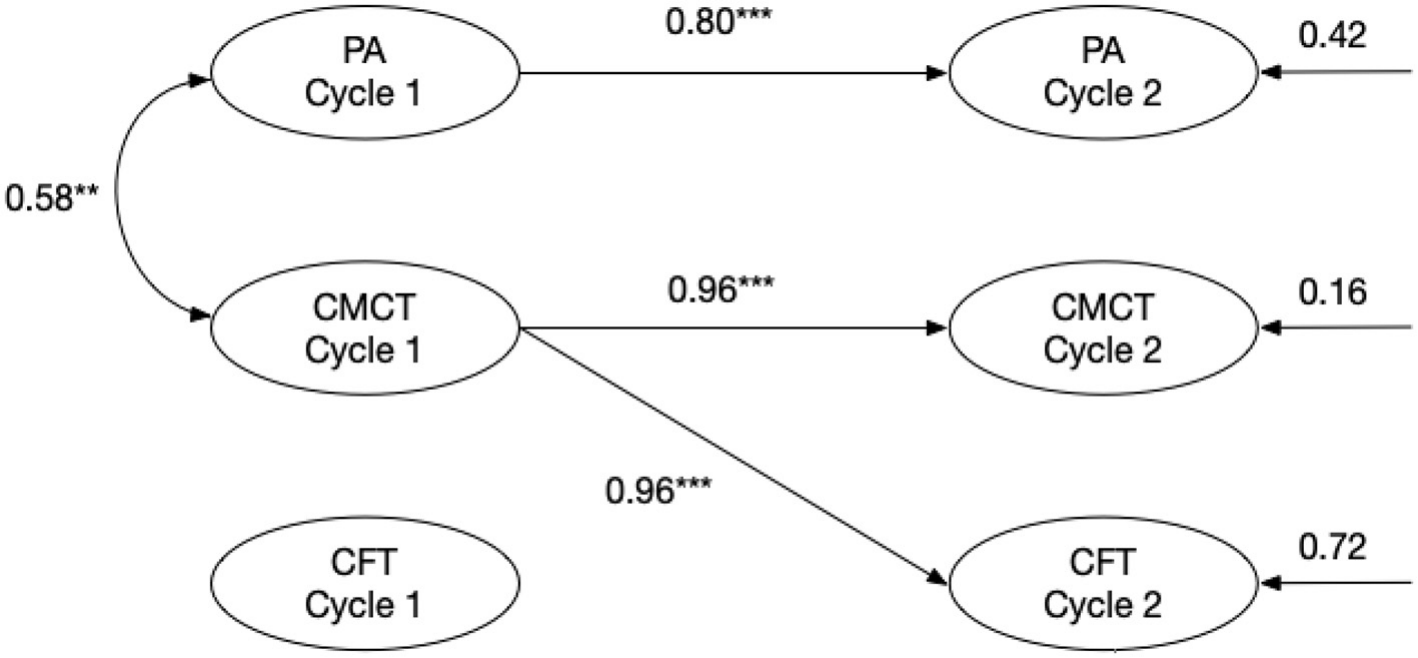
Cross-lagged model of PA, CMCT, and CFT. **p < 0.01, ***p < 0.001.

Our modeling strategy follows prior cross-lagged designs in Chinese literacy research (Lin et al., 2019). Cross-lagged analyses of phonological awareness (PA), Chinese pinyin reading (CMCT), and Chinese oral proficiency (CFT) (shown in Figure 2) demonstrated excellent model fit χ^2^(1) = 1.77, *p* > 0.40, RMSEA = 0, SRMR = 0.04, CFI = 1, TLI = 1. At Cycle 1, PA was significantly related to CMCT (β = 0.58, *p* < 0.01), but only predicted its own stability at Cycle 2 (β = 0.80, *p* < 0.001). In contrast, Cycle 1 CMCT strongly predicted both Cycle 2 CMCT (β = 0.96, *p* < 0.001) and Cycle 2 CFT (β = 0.51, *p* < 0.001). Cycle 1 CMCT did not significantly predict Cycle 2 PA (*p* > 0.05), and Cycle 1 CFT did not predict any Cycle 2 variables (*p* > 0.05). These findings indicate that CMCT performance and PA are highly stable over time, and that early CMCT performance can predict CFT 4 months later.

### 5.2 Error type analysis

#### 5.2.1 CMET error types

Three error types were found on the CMET. The first, “Task Incompletion” occurs when a participant does not recognize a word and produces no attempt despite encouragement. The second, “Form-similar Misreading” happens when a participant, unfamiliar with the whole word, recognizes some letters and substitutes it with a similar known word (e.g., misreading *queue* as *queen* or *quit* as *quiet*). The third, “Probability-based Rule Error” involves applying common phonetic patterns—such as pronouncing the *c* in *cider* as /k/ or the vowel cluster *ou* in *coud* as /əu/. Table 3 shows the frequency distribution of these error types for Schools G and N.

**Table 3:** Frequency distribution of CMET error types (percentage of total occurrences).

**Error type**	**G school**		**N school**	
	**First graders**	**Fourth graders**	**First graders**	**Fourth graders**
Error 1	4 (0.83%)	6 (1.25%)	0 (0%)	0 (0%)
Error 2	48 (10%)	33 (6.88%)	12 (2.5%)	14 (2.92%)
Error 3	95 (19.78%)	109 (22.71%)	46 (9.58%)	42 (8.75%)

In the error analysis, any mispronunciation (even if partially correct) was recorded as an error; consequently, the overall error rate is higher than that reported in Table 1, where partially correct pronunciations received partial credit. For details on the scoring criteria, please refer to the preceding section.

Chi-square tests with Fisher's exact test revealed no significant differences in error frequencies between first graders (χ^2^ = 4.135, *p* > 0.05) or fourth graders (χ^2^ = 0.301, *p* > 0.05) across the two schools. This suggests that the error types are similar, countering the idea that Chinese phonetic interference affects English pronunciation. A closer look confirmed these findings: G School's first graders had 4 error-free words, identical to N School's first graders. In fourth grade, G School had 16 error-free words, while N School had 11, with 10 words being the same, indicating a high level of homogeneity between the schools.

**Figure 3:**
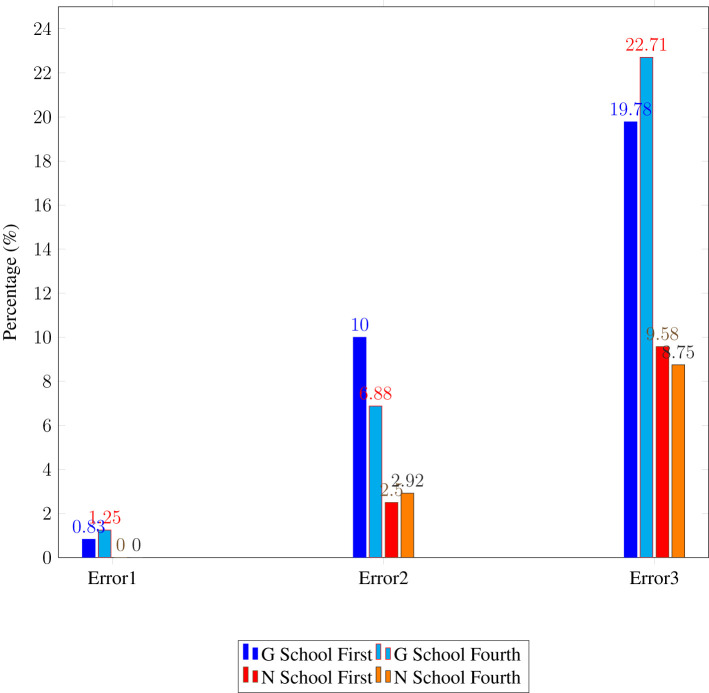
Frequency distribution of CMET error types (percentage) for first and fourth graders by school.

Figure 3 visually presents the frequency distribution of the error types across both schools and grades. As shown in the figure, the minimal differences between the schools further reinforce the statistical findings that the error patterns in English word reading are comparable across the two settings.

#### 5.2.2 CMCT error types

In School G, both first- and fourth-graders showed significant English interference when learning Chinese pinyin, often forgetting the pronunciation rules. Two main issues emerged: (1) forgetting the rules and (2) knowing the rules but failing to produce sounds that don't exist in English.

Drawing on classroom teaching experience and empirical test results, it was observed that the majority of participants correctly produced the pinyin of b, p, m, f, d, t, n, l, g, k, h, r, ch, sh, y, w, a, i, u, ai, ei, ie, an, en, in, ing, iao, ia, ian, ua, and ong. However, errors occurred more frequently on pinyin's finals of o, e, ü, üe, er, un, uo, ün, ang, eng, iong, and uang, as well as on the initials of j, q, x, z, c and zh, errors that were primarily attributable to lapses in recalling the appropriate pronunciation rules. Furthermore, for o, ü, üe, er, iang, un, uai, uan, üan, ün, ong, q, x and z, participants generally failed to acquire the correct articulatory methods, resulting in inaccurate productions.

Students also tended to add an extra /n/ to pre-nasal syllables (like an and en) and a /g/ to post-nasal syllables (like ing, eng, and ong) in accordance with English pronunciation habits. Our observed /n/ and /g/ insertions align with cross-code invented-spelling patterns documented in multilingual first graders (Zhou and McBride, 2023). Additionally, for certain post-nasal syllables such as ang and ong, the presence of analogous English words (e.g., *bang* and *long*) led students to produce pronunciations resembling /bæn/ and /lʌŋ/, respectively.

These results highlight the complex interplay between native language interference and learning Chinese pinyin in an English-speaking context.

### 5.3 Correlation analysis of test scores

A sample of 16 first-grade and 16 fourth-grade students from G School was selected to investigate the relationships among nonverbal NA, PA, PPVT-4, CMET, CFT and CMCT using bivariate correlation analysis. For first-grade students, CMCT scores were positively correlated with NA, PA, and CMET scores, whereas the correlation between CMCT and CFT scores was not statistically significant. In contrast, among fourth-grade students, CMCT scores were only positively correlated with CMET and CFT scores.

**Figure 4:**
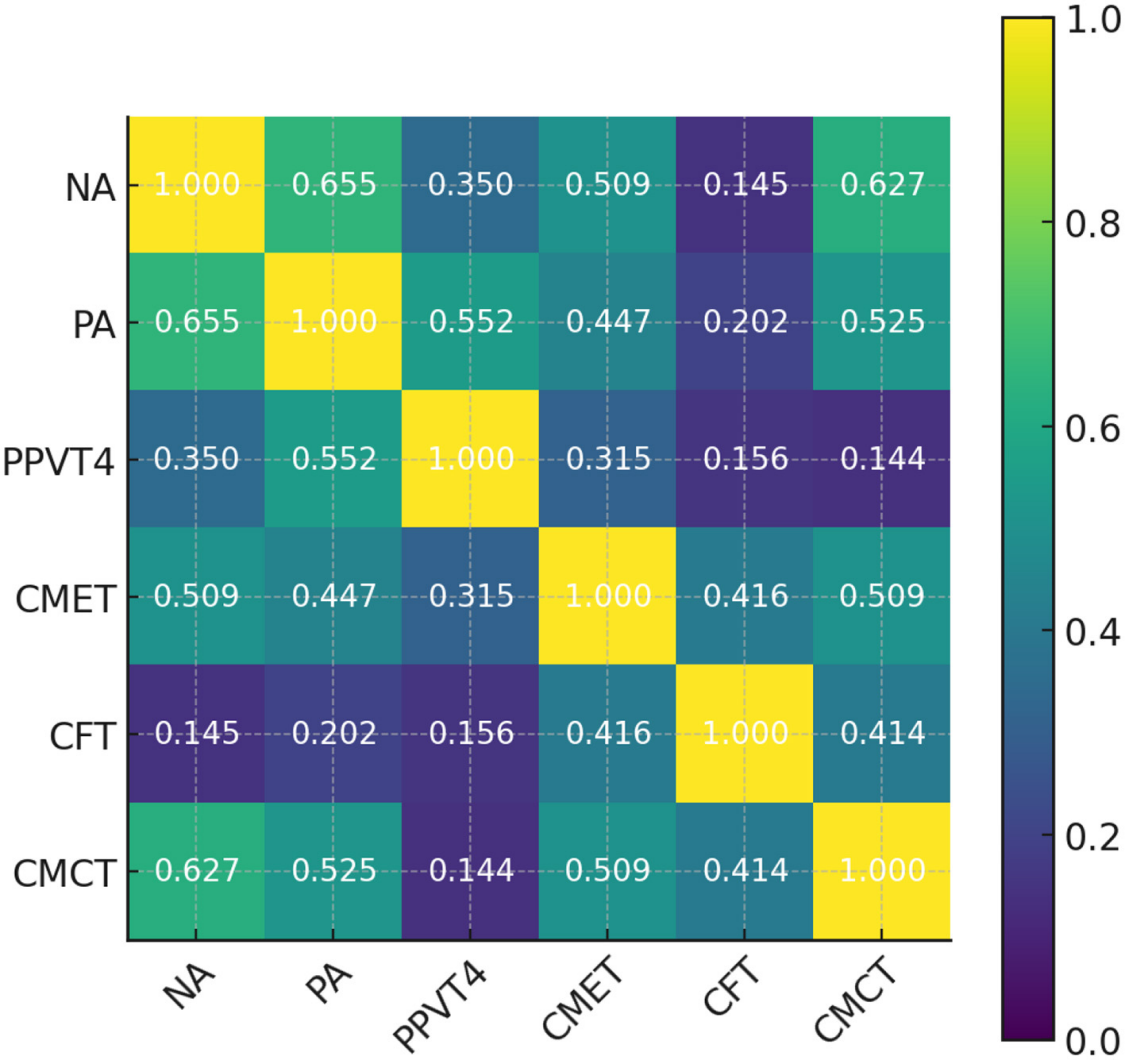
Heatmap of correlation coefficients for first-grade test scores at G school.

**Figure 5:**
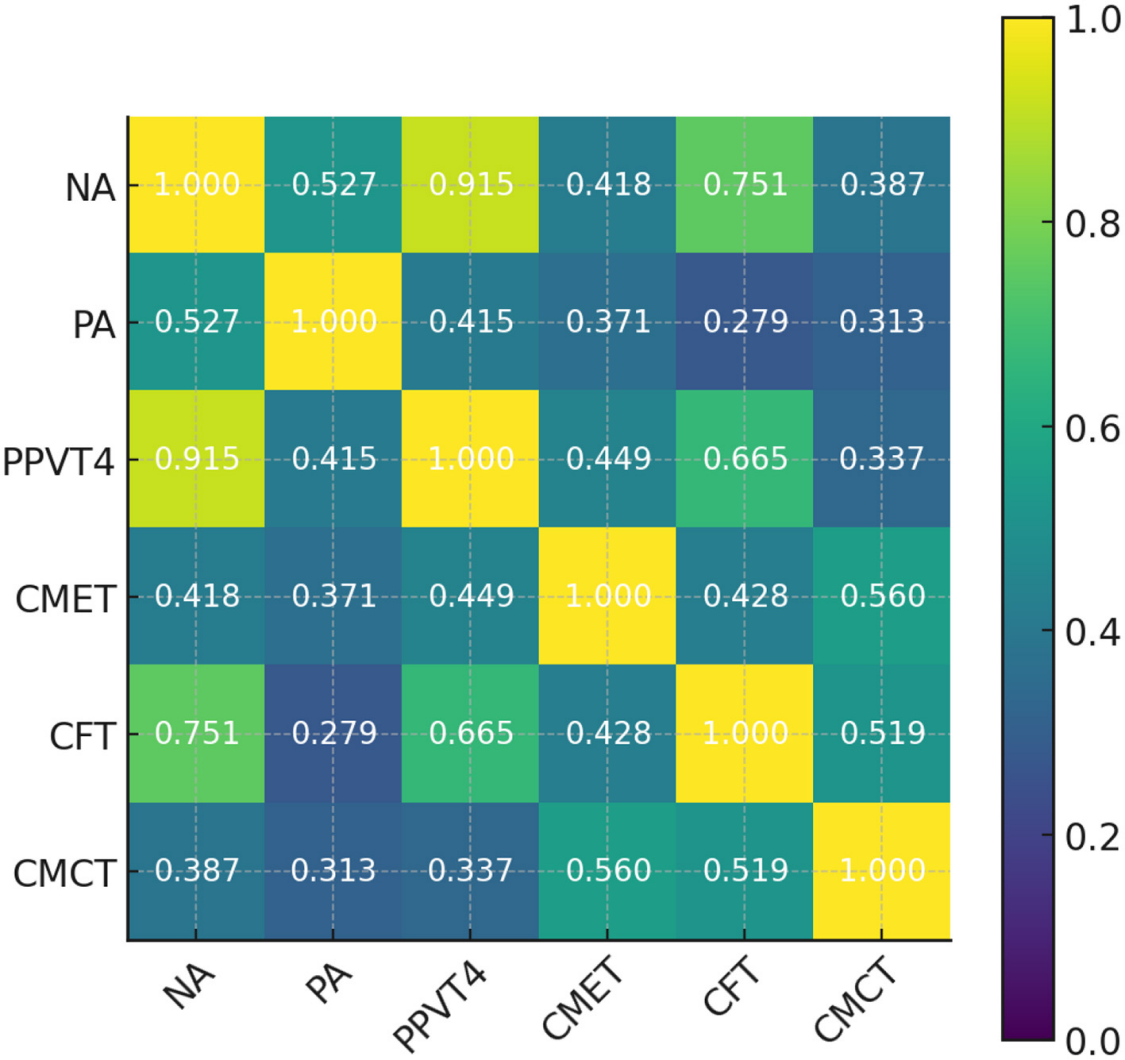
Heatmap of correlation coefficients for fourth-grade test scores at G school.

The correlation analyses, as summarized in Tables 4, 5, and visually depicted in Figures 4, 5, reveal distinct patterns across grade levels at G School. Among first-grade participants, CMCT scores exhibited statistically significant positive correlations with NA, PA, and performance on CMET, whereas the association with CFT was not significant. In contrast, for fourth-grade students, CMCT scores were significantly correlated only with CMET and CFT scores, with no significant relationships observed with NA or PA. These differential patterns suggest that a developmental shift in how Chinese pinyin interacts with students' cognitive and linguistic abilities across grade levels. Specifically, at early stage, pinyin knowledge is not yet robust enough to support fluent spoken Chinese; by fourth grade, students have likely internalized basic phonological and cognitive processing skills, and pinyin proficiency becomes more functionally tied to actual language performance.

**Table 4:** Correlation analysis of test scores for first-grade students at G school.

	**NA**	**PA**	**PPVT-4**	**CMET**	**CFT**	**CMCT**
NA		0.655^**^	0.350	0.509^*^	0.145	0.627^**^
PA	0.655^**^		0.552^*^	0.447	0.202	0.525^*^
PPVT-4	0.350	0.552^*^		0.315	0.156	0.144
CMET	0.509^*^	0.447	0.315		0.416	0.509^*^
CFT	0.145	0.202	0.156	0.416		0.414
CMCT	0.627^**^	0.525^*^	0.144	0.509^*^	0.414	

^**^Correlation is significant at the 0.01 level (two-tailed); ^*^Correlation is significant at the 0.05 level (two-tailed).

**Table 5:** Correlation analysis of test scores for fourth-grade students at G school.

	**NA**	**PA**	**PPVT-4**	**CMET**	**CFT**	**CMCT**
NA		0.527^*^	0.915^**^	0.418	0.751^**^	0.387
PA	0.527^*^		0.415	0.371	0.279	0.313
PPVT-4	0.915^**^	0.415		0.449	0.665^**^	0.337
CMET	0.418	0.371	0.449		0.428	0.560^*^
CFT	0.751^**^	0.279	0.665^**^	0.428		0.519^*^
CMCT	0.387	0.313	0.337	0.560^*^	0.519^*^	

^**^Correlation is significant at the 0.01 level (two-tailed); ^*^Correlation is significant at the 0.05 level (two-tailed).

This shift reflects that the role of pinyin learning evolves with age: in younger children, it is scaffolded by broader cognitive and phonological skills; in older children, it supports more advanced language functions. It may also reflect a decrease in native-language (English) interference and an increase in the integration of pinyin into broader Chinese language proficiency. Neuro-developmental evidence shows restructuring of cortical reading networks across Grades 1–4 (Cao, 2023), dovetailing with our behavioral shift from PA-linked to lexicon-linked Pinyin effects.

As for why the interference of the native language (English) on pinyin learning diminishes, and how pinyin is affected by phonological awareness, these issues will be analyzed in detail in the following sections.

## 6 Discussion of experimental results

### 6.1 The impact of pinyin learning on English words acquisition

After controlling for NA factors, first- and fourth-grade samples were chosen to compare PPVT-4 and CMET performance between two schools. The analysis showed no significant score differences for either grade. Notably, even with pinyin instruction, G School participants did not exhibit the predicted interference with English reading. This suggests that the limited Chinese instruction (five 30-min sessions per three weeks) offers only modest pinyin exposure, which does not disrupt English word acquisition.

In contrast to French or German, English typically exhibits relatively fixed rules for the pronunciation of letters and letter combinations, with few exceptions. However, many English letters or letter combinations do not follow consistent patterns. Consequently, it is appropriate—and indeed expected—for first-grade American children to apply their existing knowledge of these pronunciation rules when attempting to read unfamiliar words, and errors in pronunciation are therefore common. Nonetheless, even under these circumstances, the G School participants did not show any evidence that their exposure to Chinese pinyin—whose pronunciation rules differ from those of English—impeded the development of their ability to read English words.

A detailed look at error frequencies and types in the CMET revealed similar patterns across both schools, with no significant differences. There was no indication that Chinese pronunciation rules affected English reading; most errors were due to intrinsic properties of English. Only two errors appeared pinyin-influenced: some read *tongue* with a /g/ sound in the end and *coud* with a /əu/ sound in the middle. Both schools had comparable error rates (15.63% for *tongue* and roughly 15.63% at G School vs. 12.5% at N School for *coud*), suggesting these issues primarily stem from English characteristics—such as the common /g/ sound in words like *tag* and *glory* and the low-frequency nature of *coud* resembling *cold*.

Further, Error Type 2, “form-similar misreading” occurs when unfamiliar words are replaced by similar-known ones. For example, *quit* was often read as /qwaiət/ and *suit* as /sit/ or /swi:t/, reflecting similarities with words like *quiet* or *sweet*. This substitution strategy in elementary readers explains analogous errors during pinyin reading.

To complement the quantitative findings, we also examined qualitative evidence from classroom transcripts and teacher notes. For example, one first-grade student at School G pronounced the English word tongue as /tʌŋɡ/, inserting a final /ɡ/ sound. Although this error superficially resembles the influence of Chinese pinyin (where -nɡ is pronounced with a velar nasal), teacher interviews indicated that such patterns were consistent with early English reading strategies observed in monolingual peers. Similarly, several students misread queue as queen, which reflects reliance on orthographic similarity rather than interference from pinyin. These case examples align with our statistical analyses, reinforcing the interpretation that most observed errors stem from intrinsic characteristics of English orthography and phonology rather than negative transfer from pinyin instruction.

### 6.2 Benefits of learning pinyin

After controlling for NA, first-grade experimental students scored significantly higher on PA than controls. In first grade, CMCT correlated with NA, PA, and CMET but not with CFT; in contrast, fourth-grade CMCT scores correlated only with CMET and CFT. These findings imply that the most effective period for introducing pinyin instruction appears to be around the first grade, when phonological systems are still developing. By fourth grade, when these systems are largely consolidated, the cognitive benefits of pinyin learning become less direct, although early pinyin skills then appear to support vocabulary acquisition.

Second, cross-lagged analysis in the experimental group showed that at the start of learning, both types of confusable words were strongly linked to the English phonological awareness. However, 4 months later, only CMCT performance and PA predicted CFT. This finding suggests that while learning CMCT helps develop PA, its direct impact on CFT comes from the combination of PA and CMCT skills. Notably, even though reading performance on CMCT did not improve between semesters, it still predicted later CFT. This implies that measurable gains in reading may take longer, but the learning process itself alters the children's mental lexicon. In essence, foreign language learning may not yield immediate progress, yet it leaves lasting traces that gradually enhance children's ability to distinguish subtle differences in written forms and sounds, thereby bolstering PA.

### 6.3 Pinyin proficiency in the experimental group

The experimental group's pinyin proficiency remains suboptimal; participants frequently forget Chinese pinyin pronunciation rules and often confuse them with English phonetic rules. This unidirectional interference suggests that negative transfer from English adversely affects the acquisition of Chinese pinyin, thereby impeding the retention and recall of the pinyin system.

Students also make cross-linguistic errors with ambiguous pinyin words. For example, some pronounce *he* as /hi:/ due to English influence. With pre-nasal syllables like *an* and *en* an extra /n/ is often added, and with post-nasal syllables such as *ing, eng*, and *ong*, an extra /ɡ/ is common. Additionally, similar-sounding English words (e.g., *bang* or *long*) lead to errors like /bæn/ and /lʌŋ/. While the first two errors, similar to those seen in Spanish learners of English (e.g., producing forms like *estop* or *esky*), need only occasional reminders, the latter clearly result from negative transfer and may become entrenched if not promptly addressed.

The observed developmental shift—from general cognitive and phonological predictors in Grade 1 to lexical predictors in Grade 4—can be interpreted through existing developmental models of reading. For example, Ziegler and Goswami's grain size theory (2005) argues that early readers rely primarily on small phonological units (e.g., phonemes and syllables), whereas more advanced readers increasingly draw on larger units such as rimes, morphemes, and whole-word representations. This framework helps explain why first graders in our study showed strong links between pinyin performance and phonological awareness, while fourth graders demonstrated associations between pinyin and vocabulary knowledge. As children's reading systems consolidate, their reliance shifts from broad cognitive scaffolds to more language-specific lexical processing. In this sense, pinyin instruction may serve different developmental functions: initially reinforcing general phonological awareness, and later facilitating access to lexical-semantic representations in Chinese.

While the grain size theory provides one framework for interpreting the observed developmental shift, alternative explanations should also be considered. One possibility is that the shift reflects differences in instructional focus across grades. In Grade 1, Chinese instruction emphasized basic sound-symbol correspondences and phonological skills, which may explain why pinyin performance correlated strongly with phonological awareness. By Grade 4, the curriculum placed more emphasis on vocabulary building and sentence-level comprehension, making lexical factors more salient. Another possibility is that the shift is partly due to maturation effects unrelated to pinyin instruction. As children grow older, their phonological systems and general cognitive capacities consolidate, leading them to rely more on lexical-semantic processing in both their first and second languages. From this perspective, the observed Grade 4 patterns may reflect developmental trajectories common to bilingual literacy acquisition, rather than a direct effect of pinyin per se. These alternative explanations suggest that developmental changes in predictors of language outcomes are likely multifactorial, arising from the interaction of instructional design, cognitive maturation, and cross-linguistic transfer.

## 7 Conclusions

In conclusion, the findings of this study indicate that the limited amount of Chinese instruction provided in American elementary schools—specifically, five 30-min classes per three weeks, results in only a modest level of pinyin instruction that does not significantly interfere with students' ability to read English words acquisition, as evidenced by comparable performance on PPVT-4 and CMET across groups. While the native English phonological system does exert a measurable degree of interference on the acquisition of Chinese pinyin, this phenomenon reflects a typical pattern of native language dominance over a less-established foreign language. The experimental results are consistent with similar studies (Kenner et al., 2004), indicating that the phenomenon of strong native language dominance and weak foreign language proficiency is somewhat universal in children's foreign language learning. Such substitution errors mirror documented cross-lexical interference patterns in bilingual phonetic production (Amengual, 2016).

Moreover, the study reveals that Chinese pinyin instruction confers notable benefits on language development. For first-grade students, pinyin learning enhances phonological awareness, whereas for fourth-grade students, it supports the acquisition of Chinese vocabulary. In both cases, improved pinyin proficiency appears to bolster students' overall self-confidence when encountering new linguistic material, enabling them to leverage existing language knowledge in problem-solving.

Overall, these results suggest that, within the constraints of the current instructional schedule, pinyin learning in American elementary schools is beneficial and does not compromise English literacy. Importantly, with the rise of digital pinyin input as a literacy scaffold (Luo et al., 2023), future studies should further investigate how digital tools can be systematically integrated into bilingual education. For instance, keyboard-based input tasks, gamified learning apps, or interactive online platforms may not only consolidate grapheme–phoneme mappings in authentic digital environments but also increase learner engagement and sustainability. Such digital scaffolds are playing an increasingly important role in bilingual learning contexts, and their effective integration with traditional classroom instruction warrants further empirical exploration. That said, the relatively small sample size (*N* = 64) and narrow geographic scope of this study inevitably limit the generalizability of its findings. Future research should therefore replicate this work on a larger scale and across more diverse educational settings to validate the observed patterns and to further refine bilingual education practices. In addition, future research should examine the long-term effects of these digital interventions and explore strategies to mitigate negative transfer from native language phonology, with the ultimate goal of optimizing bilingual education curricula.

This study investigated how learning Chinese pinyin influences American elementary school children who are native English speakers. Two groups of students were compared: one received Chinese instruction with pinyin, while the other had no Chinese exposure. The study contributes to the field in three key ways. First, it examines the effects of limited, school-based exposure to pinyin—a “low-dose” context typical of U.S. elementary programs—rather than immersion or adult learning, thereby testing whether stage-specific benefits can emerge without intensive instruction. Second, it theorizes and empirically tests a developmental shift from phonological to lexical correlates, refining accounts of cross-linguistic transfer by highlighting the roles of timing and developmental readiness. Third, by integrating group comparisons with stability and cross-lagged analyses, it demonstrates that pinyin can foster early phonological skills without undermining English literacy, thus extending bilingual education theories to minimal-exposure contexts.

## Data Availability

The raw data supporting the conclusions of this article will be made available by the authors, without undue reservation.
