# Supplementary material for: The cognitive impacts of Chinese pinyin learning on English-speaking elementary students in a bilingual educational setting
Source: Front Psychol. 2025 Oct 27;16:1626414. doi: 10.3389/fpsyg.2025.1626414 (PMC12597923; doi:10.3389/fpsyg.2025.1626414)
Supplement: Supplementary file 1 [file Data_Sheet_1.pdf]

Test Item Examples of CMET, CFT and CMCT

1 COMMONLY MISPRONOUNCED ENGLISH WORDS READ ALOUD TEST (CMET)

Participants were asked to read aloud a series of printed English words. The test comprised 30 words that were selected based on their frequency in the English lexicon and their susceptibility to interference from Chinese phonological patterns. The word list included: long, pan, hang, sun, suit, flunk, noun, route, tongue, Cajun, pixie, pong, lunatic, coud, cede, cat, total, elephant, dare, poem, quit, cider, lawn, owl, remake, queue, monetary, woe, Bangkok, and shank. The first 15 words were chosen because their pronunciation at the letter (alphabetic) level is particularly vulnerable to the influence of Chinese pinyin instruction, while the subsequent 15 words were selected due to their potential to be affected at the syllabic level (Khanal et al., 2021; Lin, 2007).

2 CHINESE FLUENCY TEST (CFT)

The Chinese Fluency Test (CFT) was administered using a translation task in which participants listened to the examiner read aloud Chinese words, phrases, and sentences, and then immediately provided their corresponding English translations. For Grade 1 students, the test battery comprised three components: a list of 24 Chinese words, 3 Chinese phrases, and 3 Chinese sentences. The 24 words were as follows:

- **kāfēisè** (coffee-colored)
- **lánsè** (blue)
- **nǚháizi** (girl)
- **gāogāoxìngxìng** (happily)
- **mǎmǎhūhū** (so-so)
- **hóuzi** (monkey)
- **shé** (snake)
- **è** (hungry)
- **mótuōchē** (motorcycle)
- **duìbùqǐ** (sorry)
- **xièxie** (thank you)
- **yéye** (grandfather)
- **gǎnlǎnqiú** (rugby/football)
- **bízi** (nose)
- **lǎoshī** (teacher)
- **xǐhuan** (like)
- **tāmen** (them)
- **làbǐ** (crayon)
- **diànshì** (television)
- **méiyǒu** (none)
- **shǎndiàn** (lightning)

- **qíngtiān** (sunny day)
- **chènshān** (shirt)
- **Měiguó** (the United States of America)

The three phrases were:

- **sānshíliù** (thirty-six)
- **qībǎi sìshíyī** (seven hundred and forty-one)
- **Sūnwùkōng** (Sun Wukong)

The three sentences were:

- **Nǐ hǎo ma?** (How are you?)
- **Nǐ jiào shénme míngzì?** (What is your name?)
- **Tiānqì zěnmeyàng?** (How is the weather?)

For Grade 4 students, the same 24-word list was administered; however, the subtests on phrases and sentences were expanded. Specifically, the Grade 4 version included 6 Chinese phrases—namely:

- **sāndiǎnbàn** (half past three)
- **yījiǔbāwǔ nián** (the year 1985)
- **sānbǎi liùshísì** (three hundred and sixty-four)
- **nǐ de gǒu** (your dog)
- **wèishénme** (why)
- **chī jīròu** (eat chicken)

as well as 6 Chinese sentences:

- **Nǐ jiào shénme míngzì?** (What is your name?)
- **Nǐ cóng nǎlǐ lái?** (Where are you from?)
- **Duōshǎo qián?** (How much is it?)
- **Wǒ ài dǎ pīngpāngqiú.** (I love playing table tennis.)
- **Tiānqì zěnmeyàng?** (How is the weather?)
- **Nǎlǐ bù shūfú?** (Where are you feeling unwell?)

This translation task is designed to assess participants' proficiency in processing Chinese lexical items and syntactic structures, thereby providing a measure of their overall fluency in Chinese.

3 COMMONLY MISPRONOUNCED CHINESE PINYIN READ ALOUD TEST (CMCT)

Participants were instructed to read aloud a series of printed Chinese pinyin syllables. The test items were selected from syllables that children had previously encountered in their Chinese language instruction and that are particularly prone to being confused with English pronunciation. A total of 35 words were included in the test:

yi, yu, wo, er, ge, me, qi, he, nü, zi, qu, za, chi, fan, lan, cai, zhai, nian, bang, huang, yao, wei, xie, shen, leng, sun, lun, cou, rou, liu, yue, you, shui, jing, long.

Importantly, none of these items were annotated with tone markers in order to avoid providing cues that might lead to pronunciation differences relative to English. This omission was intended to ensure that the test specifically measured the influence of Chinese pinyin learning on the pronunciation of the syllables, without the confounding effects of tonal information (Bassetti, 2007; Dong, 2023).

REFERENCES

Bassetti, B. (2007). Effects of hanyu pinyin on pronunciation in learners of chinese as a foreign language. In *The Cognition, Learning and Teaching of Chinese Characters*, eds. A. Guder, X. Jiang, and e. Y. Wan (Beijing, China: Beijing Language and Culture University Press). 155–179

Dong, S. (2023). The influence of pinyin on english learning and the way to learn phonetic symbols correctly to avoid the adverse effect of pinyin. In *2023 7th International Seminar on Education, Management and Social Sciences (ISEMSS 2023)* (Atlantis Press), 436–442

Khanal, S., Johnson, M. T., and Bozorg, N. (2021). Articulatory comparison of l1 and l2 speech for mispronunciation diagnosis. In *2021 IEEE Spoken Language Technology Workshop (SLT)* (IEEE), 693–697

Lin, H.-Y. C. (2007). *Phonological interference between English and Chinese when learning Mandarin* (University of Kansas)
